# The choice of the intravenous fluid influences the tolerance of acute normovolemic anemia in anesthetized domestic pigs

**DOI:** 10.1186/cc11324

**Published:** 2012-04-30

**Authors:** Andreas Pape, Saskia Kutschker, Harry Kertscho, Peter Stein, Oliver Horn, Mischa Lossen, Bernhard Zwissler, Oliver Habler

**Affiliations:** 1 Clinic of Anesthesiology, Intensive Care Medicine and Pain Management, J.W. Goethe-University Hospital Frankfurt, Theodor-Stern-Kai 7, Frankfurt/Main, 60590, Germany; 2 Clinic of Anesthesiology, Ludwig Maximilians University Hospital, Marchioninistraße 15, Munich, 81377, Germany; 3 Clinic of Anesthesiology, Surgical Intensive Care Medicine and Pain Management, Krankenhaus Nordwest, Steinbacher Hohl 2-26, Frankfurt/Main, 60488 Germany

## Abstract

**Introduction:**

The correction of hypovolemia with acellular fluids results in acute normovolemic anemia. Whether the choice of the infusion fluid has an impact on the maintenance of oxygen (O_2_) supply during acute normovolemic anemia has not been investigated so far.

**Methods:**

Thirty-six anesthetized and mechanically ventilated pigs were hemodiluted to their physiological limit of anemia tolerance, reflected by the individual critical hemoglobin concentration (Hb_crit_). Hb_crit _was defined as the Hb-concentration corresponding with the onset of supply-dependency of total body O_2_-consumption (VO_2_). The hemodilution protocol was randomly performed with either tetrastarch (6% HES 130/0.4, TS-group, *n *= 9), gelatin (3.5% urea-crosslinked polygeline, GEL-group, *n *= 9), hetastarch (6% HES 450/0.7, HS-group, *n *= 9) or Ringer's solution (RS-group, *n *= 9). The primary endpoint was the dimension of Hb_crit_, secondary endpoints were parameters of central hemodynamics, O_2 _transport and tissue oxygenation.

**Results:**

In each animal, normovolemia was maintained throughout the protocol. Hb_crit _was met at 3.7 ± 0.6 g/dl (RS), 3.0 ± 0.6 g/dl (HS *P *< 0.05 vs. RS), 2.7 ± 0.6 g/dl (GEL, *P *< 0.05 vs. RS) and 2.1 ± 0.4 g/dl (TS, *P *< 0.05 vs. GEL, HS and RS). Hemodilution with RS resulted in a significant increase of extravascular lung water index (EVLWI) and a decrease of arterial oxygen partial pressure (paO_2_), and O_2 _extraction ratio was increased, when animals of the TS-, GEL- and HS-groups met their individual Hb_crit_.

**Conclusions:**

The choice of the intravenous fluid has an impact on the tolerance of acute normovolemic anemia induced by acellular volume replacement. Third-generation tetrastarch preparations (e.g., HES 130/0.4) appear most advantageous regarding maintenance of tissue oxygenation during progressive anemia. The underlying mechanism includes a lower degree of extravasation and favourable effects on microcirculatory function.

## Introduction

The correction of hypovolemia is an essential goal in the treatment of critically ill patients. However, the use of acellular fluids (that is, crystalloid or colloidal solutions) results in the dilution of the circulating cell mass (acute normovolemic anemia) with a corresponding decrease of O_2 _transport capacity [1-3].

Acute normovolemic anemia is initially compensated by increases of cardiac output and arterio-venous O_2 _extraction [4]. Moreover, O_2 _supply (DO_2_) physiologically exceeds O_2 _demand (reflected by total body O_2 _consumption (VO_2_) under resting conditions) by the factor three to four. When DO_2 _begins to decrease at lower Hb concentrations, the total body O_2 _demand is still met, and VO_2 _remains constant despite decreasing Hb concentrations (O_2 _supply-independency of VO_2_). When DO_2 _falls below a critical value, the amount of O_2 _delivered to the tissues becomes insufficient to meet their O_2 _demand and VO_2 _starts to decline (O_2 _supply-dependency of VO_2_) [5]. The corresponding critical hemoglobin concentration (Hb_crit_) represents the ultimate limit of the individual anemia tolerance.

Among clinicians all over the world, infusion practice varies considerably and different intravenous (IV) fluids are used for volume replacement [6]. However, currently available IV fluids differ in their pharmacodynamic and kinetic profile (for example, molecular weight, plasma half-life, volume expansion effect) and in their effects on rheology and microcirculatory function.

We hypothesized that these properties might have an influence on the tolerance of acute normovolemic anemia. In this regard, the present study was conceived to compare potential effects of four commonly used IV fluids: low-molecular hydroxyethyl starch (HES) (tetrastarch, 6% HES 130/0.4), high-molecular HES (hetastarch, 6% HES 450/0.7), gelatin (3.5% urea-crosslinked polygeline), and crystalloid volume replacement with Ringer's solution.

## Materials and methods

After approval by the local governmental review board (Regional Council Darmstadt, department for veterinary affairs/V54), experiments were performed in 36 healthy farm-bred pigs of either sex (body weight 24.0 ± 3.7 kg). All animals received good care in compliance with the Guide for the Care and Use of Laboratory Animals.

### Anesthesia and ventilation

Animals were denied food 12 hours before each experiment started, but had free access to water. After intramuscular premedication with 10 mg/kg ketamine (Ketavet™, Parke-Davis, Berlin, Germany) and 1 mg/kg midazolam (Midazolam™, Ratiopharm, Ulm, Germany), anesthesia was induced by IV injection of 3 mg/kg propofol (Propofol™, Braun, Melsungen, Germany) and 30 μg/kg fentanyl (Fentanyl™, Janssen, Neuss, Germany) and maintained by continuous infusion of propofol (0.16 mg/kg/min), midazolam (0.01 mg/kg/min) and fentanyl (0.8 μg/kg/min). To facilitate ventilation, muscular paralysis was achieved with pancuronium bromide (Pancuronium™, DeltaSelect, Dreieich, Germany, bolus injection 0.2 mg/kg after induction of anesthesia, followed by continuous infusion of 0.13 mg/kg/min). Estimated fluid losses were replaced with Ringer's solution (Ringer-Infusionslösung™, Braun, Melsungen, Germany, 3 mL/kg/h).

Animals were orotracheally intubated and ventilated with ambient air at a rate of 14 cycles/min and a positive end-expiratory pressure of 5 cmH_2_O (Servo 900B, SiemensElema, Solna, Sweden). Tidal volume was individually adjusted to provide arterial normocapnia and was then maintained throughout the entire protocol.

### Instrumentation and monitoring

Animals were placed in the supine position and a five-lead electrocardiogram (II, V5) was installed for detection of arrhythmias and ST-segment changes. A double-lumen catheter (Arrow, Reading, PA, USA) was inserted into the cranial vena cava and a Swan-Ganz-Catheter (Baxter, Irvine, CA, USA) was floated into a branch of the pulmonary artery. Each one 6F introducer sheath (Arrow, Reading, PA, USA) was inserted into the right femoral vein and artery, respectively. For continuous measurement of arterial blood pressure and cardiac output, a thermodilution catheter was placed into the left femoral artery (Pulsion Medical Systems, Munich, Germany). Body core temperature was kept constant using a warming pad.

### Experimental protocol

Upon completion of catheter insertion and installation of the different measuring devices, a 60 min stabilization period was allowed to elapse before the first data set was recorded (baseline). Subsequently, animals were randomized to undergo acute normovolemic hemodilution with one of the following fluids: 1) 6% HES 130/0.4 (tetrastarch, TS-group, *n *= 9); 2) 6% HES 450/0.7 (hetastarch, HS-group, *n *= 9); 3) 3.5% urea-crosslinked polygeline (gelatin, GEL-group, *n *= 9), or 4) Ringer's solution (RS-group, *n *= 9).

Acute normovolemic anemia was induced by withdrawal of blood (1mL/kg/min) and simultaneous infusion of one of the IV fluids. To maintain normovolemia during the hemodilution protocol, the following infusion rates were chosen with regard to the different plasma expansion effects of the fluids investigated (see Table 1): Ringer's solution 3 mL/kg/min, gelatin 1.2 mL/kg/min, tetrastarch and hetastarch 1 mL/kg/min, respectively. Infusion fluids were warmed in an immersion bath until IV infusion. For precise synchronization of blood withdrawal with the corresponding infusion rates, a bidirectional precision pump (Harvard Apparatus, Holliston, MA, USA) was used.

**Table 1:** Characteristics and trade names of intravenous fluids investigated in the present study [25].

	Tetrastarch	Hetastarch	Gelatin	Ringer's solution
Concentration (%)	6	6	3.5	n.a.
Mean molecular weight (kDa)	130	450	35	n.a.
Volume efficacy (%)	100	100	80	30
Volume effect (min)	120-180	300-360	60-180	20
Viscosity (cp)	2.1	3.9	1.7	n.a.
COP in vitro (mmHg)	36	26	25	n.a.
Trade name	Voluven™, Fresenius Kabi, Bad Homburg, Germany	HESPAN™, Braun, Irvine, CA, USA	Haemaccel™, DeltaSelect, Dreieich, Germany	Ringer-Infusions-lösung™, Braun, Melsungen, Germany

COP, colloidal oncotic pressure; n.a., not applicable.

The target parameter of the hemodilution protocol was the animal's individual critical Hb concentration (Hb_crit_), which was defined as the Hb concentration corresponding with a critical limitation of O_2 _delivery to the tissues (DO_2_) and the onset of O_2 _supply-dependency of total body O_2 _consumption-VO_2 _(see below). At the end of the hemodilution protocol, animals were killed by intracardiac injection of saturated potassium chloride solution.

### Measurements

Intravascular blood volume was determined at baseline using the "whole-blood" method of the indocyanin green (ICG) indicator dilution technique, which has already been described in detail elsewhere [7]. The pressure transducers of the cardiovascular catheters were connected with a multi-channel recorder and a personal computer (Hugo-Sachs, March-Hugstetten, Germany) for continuous measurement of hemodynamic parameters. Cardiac output was continuously measured using the pulse contour analysis (PICCO classic monitor, Pulsion Medical Systems, Munich, Germany). The volumetric preload and extravasation parameters, intrathoracic blood volume (ITBV) and extravascular lung water (EVLW), were assessed after exchange of each 10% of the circulating blood volume using the transpulmonary thermodilution technique. Arterial and mixed venous blood samples were withdrawn at baseline, after exchange of each 10% of the circulating blood volume and at Hb_crit _for blood gas analysis and assessment of Hb concentration (GEM-3000 and 682 CO-Oxymeter, Instrumentation Laboratory, Lexington, MA, USA).

### Calculated parameters

Body surface area (BSA) was calculated according to Holt [8], from body weight (BW) and a species-dependent constant:

$$BSA=k\cdot BW^{\frac{2}{3}}\left(m^{2}\right),$$

where k = 9 for the species pig.

The measured values of cardiac output (CO) and O_2 _consumption (VO_2_) were indexed to BSA:

$$CI=\frac{CO}{BSA}\;\left(\frac{l}{\min\cdot m^{2}}\right)$$

$$VO_{2}I=\frac{VO_{2}}{BSA}\;\left(\frac{ml}{\min\cdot m^{2}}\right)$$

Systemic and pulmonary vascular resistance indices (SVRI and PVRI) were calculated from standard formulae:

$$SVRI=\frac{\left(MAP-CVP\right)\cdot 79.9}{CI}\;\left(\frac{dyn\cdot s}{cm^{5}\cdot m^{2}}\right)$$

$$PVRI=\frac{\left(MPAP-PCWP\right)\cdot 79.9}{CI}\;\left(\frac{dyn\cdot s}{cm^{5}\cdot m^{2}}\right)$$

where MAP = mean arterial pressure, CVP = central venous pressure, CI = cardiac index, MPAP = mean pulmonary arterial pressure and PCWP = pulmonary capillary wedge pressure.

Arterial and mixed venous O_2 _content (CaO_2 _and CvO_2_), O_2 _delivery (DO_2_I) and O_2 _extraction ratio were calculated as follows:

$$CaO_{2}=\left[Hb\right]\cdot 1.34\cdot SaO_{2}+0.0031\cdot paO_{2}\;\left(\frac{mL}{dL}\right)$$

$$CvO_{2}=\left[Hb\right]\cdot 1.34\cdot SvO_{2}+0.0031\cdot pvO_{2}\;\left(\frac{mL}{dL}\right)$$

$$DO_{2}I=CI\cdot CaO_{2}\;\left(\frac{mL}{\min\cdot m^{2}}\right)$$

$$O_{2}-ER=\frac{CaO_{2}-CvO_{2}}{CaO_{2}}\;\left(\%\right)$$

### Determination of Hb_crit_

**Figure 1:**
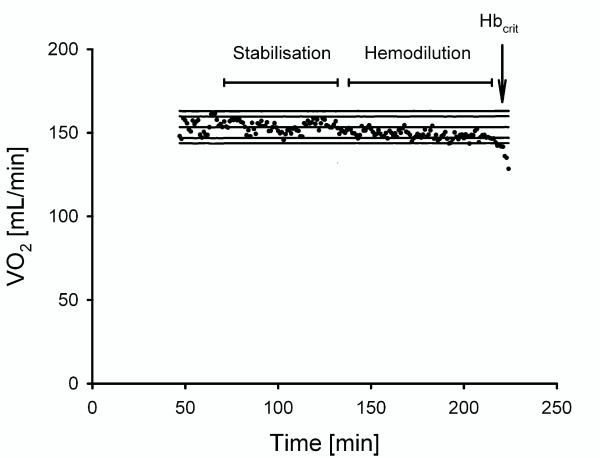
**Typical example of a recording of total body oxygen consumption (VO_2_) in the course of the experimental protocol**. A linear regression analysis including the calculation of SD was performed with VO_2 _values collected during the 60-minute stabilization period. During the subsequent hemodilution protocol, a critical limitation of oxygen delivery (DO_2_) was assumed, when three consecutive VO_2 _values fell below the lower 3σ-range.

Hb_crit _is the correlate of the critical limitation of DO_2_, and marks the onset of total body O_2 _supply-dependency. The corresponding decrease of VO_2 _was prospectively detected in an automated and investigator-independent manner: VO_2 _was measured every 60 sec with a metabolic monitor (Delta-Trac II™ MBM-200, Datex-Engstrom, Helsinki, Finland). VO_2 _values were simultaneously recorded and computed with specific software (Delta-Crit-System, DCS) [9]. During the stabilization period, the DCS enters VO_2 _values into an online regression analysis and calculates the mean and standard deviation (SD). During the subsequent hemodilution period, the VO_2 _value obtained every 60 secis compared to the mean value predicted by the DCS. When three consecutive VO_2 _values are outside the predefined range (3-fold SD of the regression line), a significant decrease of VO_2 _is assumed (see Figure 1) and the computer alerts visually and acoustically [9]. For determination of Hb_crit_, the corresponding Hb concentration was measured in the arterial blood sample.

### Statistics

Statistical analysis was performed with the SAS 9.1 software package (SAS Institute, Cary, NC, USA). All data are presented as mean ± SD. Distribution of data was assessed with the Shapiro-Wilk test. In the case of normal distribution, the time effect on the different variables, and differences between groups at different time points were tested by repeated analysis of variance (ANOVA). Post hoc analysis of differences detected with ANOVA was performed with the Student Newman Keuls test. In the case of non-normal distribution, the time effect on the parameters, and the between-group differences were tested by analysis of variance on ranks (rANOVA). Post hoc analysis of differences detected with rANOVA was performed with Tukey's test. For all parameters, statistical significance was accepted at *P *< 0.05.

## Results

### Baseline characteristics

On analysis of baseline body weight, blood volume index and all investigated parameters of hemodynamics and O_2 _transport, there were no significant differences detected between the groups (see Tables 2 and 3).

**Table 2:** Hemodynamic parameters obtained at baseline and after hemodilution to the individual Hb_crit _of the RL-, HS-, GEL- and TS-groups.

Parameter	Group	Baseline	Hb_crit _RS-group		Hb_crit _HS-group		Hb_crit _GEL-group		Hb_crit _TS-group	
BVI	TS	73 ± 6	n.a.		n.a.		n.a.		n.a.	
(ml/kg)	GEL	76 ± 9	n.a.		n.a.		n.a.		n.a.	
	HS	73 ± 3	n.a.		n.a.		n.a.		n.a.	
	RS	76 ± 8	n.a.		n.a.		n.a.		n.a.	
HR	TS	102 ± 15	115 ± 19		120 ± 16		132 ± 25	+	133 ± 29	+
(1/min)	GEL	100 ± 23	111 ± 16		114 ± 15		126 ± 15	+	n.a.	
	HS	102 ± 19	120 ± 17		121 ± 8	+	n.a.		n.a.	
	RS	111 ± 20	127 ± 35		n.a.		n.a.		n.a.	
MAP	TS	81 ± 14	88 ± 18		84 ± 18		80 ± 19		59 ± 15	
(mmHg)	GEL	88 ± 12	78 ± 14		77 ± 15		64 ± 12	+	n.a.	
	HS	88 ± 20	75 ± 13		70 ± 13	+	n.a.		n.a.	
	RS	90 ± 20	71 ± 10		n.a.		n.a.		n.a.	
MPAP	TS	19 ± 4	23 ± 4		23 ± 4		23 ± 4		23 ± 5	
(mmHg)	GEL	19 ± 3	25 ± 6	+	24 ± 5	+	26 ± 5	+	n.a.	
	HS	18 ± 3	22 ± 6	+	24 ± 4	+	n.a.		n.a.	
	RS	18 ± 3	27 ± 11	+	n.a.		n.a.		n.a.	
CI	TS	4.2 ± 0.5	6.7 ± 0.8	+	7.5 ± 1.2	+	7.7 ± 1.1	+	8.0 ± 2.8	+
(L/min/m^2^)	GEL	4.6 ± 0.8	6.7 ± 0.7	+	7.5 ± 0.5	+	7.5 ± 0.9	+	n.a.	
	HS	4.5 ± 0.5	6.7 ± 0.6	+	7.0 ± 1.1	+	n.a.		n.a.	
	RS	4.4 ± 0.8	6.7 ± 0.8	+	n.a.		n.a.		n.a.	
SVI	TS	42 ± 8	59 ± 11	+	63 ± 11	+	59 ± 11	+	57 ± 3	+
(mL/m^2^)	GEL	47 ± 7	62 ± 12	+	67 ± 10	+	61 ± 9		n.a	
	HS	45 ± 9	56 ± 7		55 ± 11		n.a		n.a	
	RS	41 ± 9	55 ± 13	+	n.a.		n.a.		n.a.	
CVP	TS	6 ± 4	7 ± 0		8 ± 3		8 ± 2		8 ± 1	
(mmHg)]	GEL	5 ± 3	8 ± 3		8 ± 3		8 ± 3		n.a.	
	HS	6 ± 3	9 ± 4		10 ± 6		n.a.		n.a.	
	RS	4 ± 2	7 ± 3	+	n.a.		n.a.		n.a.	
PCWP	TS	5 ± 3	7 ± 2		6 ± 2		7 ± 2		8 ± 2	
(mmHg)	GEL	6 ± 2	6 ± 3		7 ± 3		7 ± 3		n.a.	
	HS	4 ± 3	5 ± 2		5 ± 3		n.a.		n.a.	
	RS	4 ± 3	6 ± 3		n.a.		n.a.		n.a.	
ITBVI	TS	673 ± 65	857 ± 142	+§	856 ± 63	+	844 ± 78	+*	764 ± 177	
(mL/m^2^)	GEL	699 ± 94	818 ± 101	+	824 ± 82	+	784 ± 57	+	n.a.	
	HS	664 ± 78	829 ± 84	+§	774 ± 86	+	n.a.		n.a.	
	RS	699 ± 34	761 ± 70	+	n.a.		n.a.		n.a.	
SVV	TS	21 ± 6	13 ± 3		10 ± 3	+	11 ± 3	+*	10 ± 2	+
(%)	GEL	19 ± 5	12 ± 3		10 ± 3	+	16 ± 4		n.a.	
	HS	20 ± 6	14 ± 2	+§	13 ± 3	+	n.a.		n.a.	
	RS	17 ± 1	18 ± 7		n.a.		n.a.		n.a.	
SVRI	TS	1447 ± 342	943 ± 178	+	817 ± 212	+	751 ± 204	+	517 ± 126	+
(dyn/sec/cm^5^/m^2^)	GEL	1489 ± 433	834 ± 180	+	736 ± 181	+	603 ± 147	+	n.a.	
	HS	1490 ± 386	801 ± 236	+	590 ± 135	+	n.a.		n.a.	
	RS	1578 ± 439	871 ± 221	+	n.a.		n.a.		n.a.	

BVI, blood volume indexed to body weight (only assessed at baseline); HR, heart rate; MAP, mean arterial pressure; MPAP, mean pulmonary arterial pressure; CI, cardiac index; SVI, stroke volume index; CVP, central venous pressure; PCWP, pulmonary capillary wedge pressure; ITBVI, intrathoracic blood volume index; SVV, stroke volume variation; SVRI, systemic vascular resistance index; n.a., not applicable. *P < 0.05 vs. gelatin, # *P *< 0.05 vs. HS, § *P *< 0.05 vs. RS, +*P *< 0.05 vs. baseline.

**Table 3:** Oxygen-derived parameters investigated at baseline and after hemodilution to the individual Hb_crit _of the RL-, HS-, GEL- and TS-groups.

Parameter	Group	Baseline	Hb_crit _RS-group		Hb_crit _HS-group		Hb_crit _GEL-group		Hb_crit _TS-group	
Hb	TS	7.6 ± 0.6	3.7 ± 0.2	+	3.0 ± 0.1	+	2.7 ± 0.1	+	2.1 ± 0.4	+
(g/dL)	GEL	7.7 ± 0.5	3.6 ± 0.3	+	2.9 ± 0.2	+	2.7 ± 0.7	+	n.a.	
	HS	7.6 ± 0.4	3.7 ± 0.3	+	3.0 ± 0.6	+	n.a.		n.a.	
	RS	7.9 ± 0.5	3.7 ± 0.6	+	n.a.		n.a.		n.a.	
Temp.	TS	37.8 ± 1.3	37.5 ± 0.5		37.6 ± 0.4		37.7 ± 1.2		37.6 ± 1.2	
(°C)	GEL	37.5 ± 1.0	37.4 ± 1.2		37.3 ± 0.8		37.2 ± 1.0		n.a.	
	HS	37.6 ± 1.1	37.6 ± 1.0		37.4 ± 1.2		n.a.		n.a.	
	RS	37.7 ± 0.7	37.4 ± 0.6		n.a.		n.a.		n.a.	
paO_2_	TS	94 ± 9	95 ± 12	§	100 ± 13		98 ± 14		105 ± 22	
(mmHg)	GEL	98 ± 10	99 ± 19	§	101 ± 14		95 ± 18		n.a.	
	HS	95 ± 7	98 ± 9	§	101 ± 11		n.a.		n.a.	
	RS	100 ± 8	79 ± 10	+	n.a.		n.a.		n.a.	
paCO_2_	TS	34 ± 3	35 ± 4		34 ± 4		34 ± 4		33 ± 4	
(mmHg)	GEL	34 ± 2	35 ± 3		34 ± 4		35 ± 4		n.a.	
	HS	33 ± 4	33 ± 3		33 ± 3		n.a.		n.a.	
	RS	32 ± 3	32 ± 4		n.a.		n.a.		n.a.	
pvO_2_	TS	34 ± 3	31 ± 5		32 ± 4		31 ± 4		29 ± 4	+
(mmHg)	GEL	34 ± 2	32 ± 3		34 ± 2	#	32 ± 4		n.a.	
	HS	33 ± 4	35 ± 4		29 ± 3	+	n.a.		n.a.	
	RS	32 ± 3	34 ± 3		n.a.		n.a.		n.a.	
DO_2_I	TS	428 ± 45	367 ± 58	+	324 ± 54	+	287 ± 20	+	253 ± 84	+
(mL/min/m^2^)	GEL	478 ± 67	348 ± 51	+	328 ± 35	+	289 ± 44	+	n.a.	
	HS	455 ± 53	353 ± 22	+	303 ± 65	+	n.a.		n.a.	
	RS	487 ± 109	339 ± 69	+	n.a.		n.a.		n.a.	
VO_2_I	TS	223 ± 33	219 ± 29		203 ± 14		216 ± 34		199 ± 35	
(mL/min/m^2^)	GEL	219 ± 17	210 ± 10		201 ± 12		199 ± 12		n.a.	
	HS	229 ± 37	199 ± 10		211 ± 35		n.a.		n.a.	
	RS	222 ± 23	203 ± 26		n.a.		n.a.		n.a.	
O_2_-ER	TS	37 ± 5	46 ± 11		45 ± 7	#	46 ± 11		52 ± 13	+
(%)	GEL	35 ± 5	46 ± 8		41 ± 7	#	45 ± 12	+	n.a.	
	HS	38 ± 9	44 ± 6		52 ± 9	+	n.a.		n.a.	
	RS	39 ± 7	44 ± 6		n.a.		n.a.		n.a.	
pH	TS	7.57 ± 0.04	7.53 ± 0.04		7.52 ± 0.04		7.51 ± 0.04		7.50 ± 0.06	
	GEL	7.58 ± 0.02	7.50 ± 0.03	+	7.49 ± 0.03		7.47 ± 0.04		n.a.	
	HS	7.59 ± 0.04	7.55 ± 0.05	+	7.53 ± 0.04		n.a.		n.a.	
	RS	7.58 ± 0.05	7.42 ± 0.08	+	n.a.		n.a.		n.a.	
BE	TS	8.7 ± 3.4	6.1 ± 3.5	§	5.0 ± 3.4		4.2 ± 3.4	+	2.4 ± 4.0	+
(mmol/L)	GEL	9.4 ± 2.0	4.0 ± 1.7	+§	2.7 ± 2.1	+	1.8 ± 2.8	+	n.a.	
	HS	9.3 ± 2.4	6.2 ± 3.0	+§	4.6 ± 2.5	+	n.a.		n.a.	
	RS	7.5 ± 2.1	-3.5 ± 3.4	+	n.a.		n.a.		n.a.	
Lactate	TS	1.9 ± 0.9	1.7 ± 0.8		1.7 ± 0.8		1.7 ± 0.9		2.3 ± 1.0	
(mmol/L)	GEL	1.4 ± 0.3	1.2 ± 0.3		1.1 ± 0.3		1.1 ± 0.3		n.a.	
	HS	1.7 ± 0.7	1.6 ± 0.5		1.8 ± 0.1		n.a.		n.a.	
	RS	1.4 ± 0.6	1.3 ± 0.6		n.a.		n.a.		n.a.	

Temp, body core temperature; Hb, haemoglobin concentration; paO_2_, arterial O_2 _partial pressure; pvO_2_, mixed venous O_2 _partial pressure; paCO_2_, arterial CO_2 _partial pressure; VO_2_I and DO_2_I, indices of O_2 _consumption and delivery; O_2_-ER, O_2 _extraction ratio; pH, pH value; BE, base excess; lactate, lactate concentration; n.a., not applicable. *P < 0.05 vs. gelatin, #*P *< 0.05 vs. HS, §*P *< 0.05 vs. RS, +*P *< 0.05 vs. baseline.

### Primary endpoint: critical Hb concentation (Hb_crit_)

**Figure 2:**
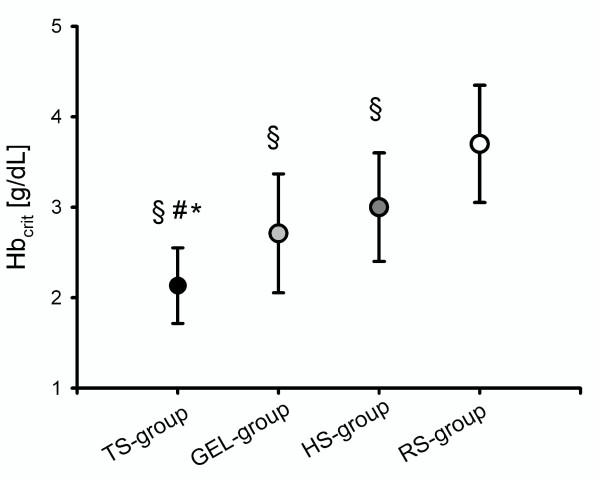
**Critical hemoglobin concentration (**_crit _**obtained after induction of acute anemia**. Anemia was induced using tetrastarch (TS-group, Hb_crit _2.1 ± 0.4 g/dL), gelatin (GEL-group, Hb_crit _2.7 ± 0.6 g/dL), hetastarch (HS-group, Hb_crit _3.0 ± 0.6 g/dL) or Ringer's solution (RS-group, Hb_crit _3.7 ± 0.6 g/dL). *P < 0.05 vs. GEL, #*P *< 0.05 vs. HS, §*P *< 0.05 vs. RS.

Depending on the plasma substitute used for hemodilution, Hb_crit _was met at 2.1 ± 0.4 g/dL (TS-group), 2.7 ± 0.6 g/dL (GEL-group), 3.0 ± 0.6 g/dL (HS-group) and 3.7 ± 0.6 g/dL (RS-group). The lowest value of Hb_crit _was attained in the TS-group (*P *< 0.05 vs. GEL, HS and RS). While Hb_crit _did not differ significantly between the GEL- and HS-groups, the difference between the RS-group and the other groups (GEL-, HS- and TS- groups) was statistically significant (see Figure 2).

The induction of critical normovolemic anemia required the exchange of 69 ± 21% of the circulating blood volume (BV) for Ringer's solution, 71 ± 19% BV for hetastarch, 93 ± 44% BV for gelatin (*P *< 0.05 vs. HS- and RS-groups) and 107 ± 28% BV for tetrastarch (*P *< 0.05 vs. HS- and RS-groups). For maintenance of normovolemia, animals received 1,812 ± 535 mL tetrastarch, 2,016 ± 898 mL gelatin, 1,369 ± 324 mL hetastarch (*P *< 0.05 vs. TS-group), or 3,698 ± 1320 mL Ringer's solution, respectively (*P *< 0.05 vs. TS- GEL- and HS-groups).

### Secondary endpoints: hemodynamic and O_2_-derived parameters

In Tables 2 and 3, hemodynamic and O_2_-derived parameters are presented at the respective critical Hb concentrations, that is, after hemodilution to 3.7 g/dL, 3.0 g/dL, 2.7 g/dL and 2.1 g/dL, respectively.

#### Hemodilution to Hb 3.7 g/dL (Hb_crit _of the RS-group)

**Figure 3:**
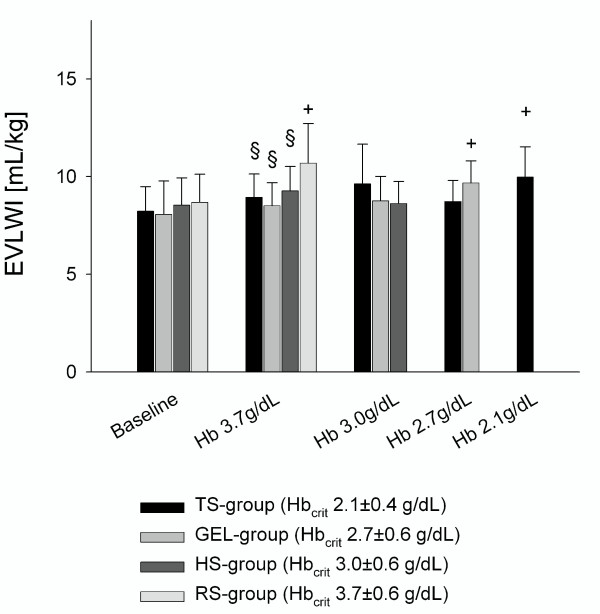
**Extravascular lung water index (EVLWI) obtained at baseline after hemodilution to Hb 3**.7 g/dL (Hb_crit _of the RS-group), Hb 3.0 g/dL (Hb_crit _of the HS-group), Hb 2.7 (Hb_crit _of the GEL-group) and Hb 2.1 (Hb_crit _of the TS-group). At Hb 3.7 g/dL, EVLWI was significantly higher in the RS-group than in the TS-, GEL-, and HS-groups. *P < 0.05 vs. GEL, #*P *< 0.05 vs. HS, §*P *< 0.05 vs. RS, +*P *< 0.05 vs. baseline. Hb_crit_, critical haemoglobin concentration, error bars represent standard deviation of mean.

As compensation for hemodilution, cardiac index (CI) increased by 46 to 60% in all groups (*P *< 0.05 vs. baseline). Consistently, stroke volume index (SVI) and heart rate (HR) increased by 24 to 40% and 14 to 18%, respectively. While the increases in SVI were significant in the TS-group, GEL-, and RS-groups, the increase in HR was not significant in any group. Mean arterial pressure (MAP) decreased by 11 to 21% in the GEL-, HS- and RS-groups (non-significant) and systemic vascular resistance index (SVRI) decreased in all groups by 35 to 46% (*P *< 0.05 vs. baseline), while mean pulmonary arterial pressure (MPAP) increased by 22 to 50% (*P *< 0.05 vs. baseline in the GEL-, HS and RS-group). Stroke volume variation (SVV) remained unchanged in the RS-group and tended to decrease in the TS- and GEL-groups. In the HS-group, the decrease in SVV was statistically significant. Compared with baseline, ITBV index (ITBVI) increased in all groups (*P *< 0.05), reflecting increased venous return to the heart. The ITBVI was higher in animals hemodiluted with TS or HS than after fluid replacement with RS (*P *< 0.05). As a consequence of hemodilution, DO_2_I decreased by 14 to 30% in all groups (*P *< 0.05 vs. baseline). Moreover, the pH-value and base excess (BE) decreased significantly in the GEL-, HS- and RS-groups. As the protocol was terminated at the onset of the critical DO_2_, lactate concentration was not yet elevated at this time point (see Table 3). The EVLW index (EVLWI) was significantly increased in the RS-group (*P *< 0.05 vs. baseline and *P *< 0.05 vs. GEL-, HS- and TS-groups, see Figure 3). Consistently, arterial oxygen partial pressure (paO_2_) was decreased in the RS-group (*P *< 0.05 vs. baseline and *P *< 0.05 vs. GEL-, HS- and TS-groups).

#### Hemodilution to Hb 3.0 g/dL (Hb_crit _of the HS-group)

As the limit of anemia tolerance was already met at Hb 3.7 g/dL in the RS-group, hemodilution to 3.0 g/dL could only be performed in the HS-, GEL- and TS-groups.

A slight increase in CI, HR and SVI and a decrease in MAP and SVRI was observed in these groups, and SVV was significantly reduced compared with baseline. The ITBVI did not change further, but remained elevated above the baseline level (*P *< 0.05). There were no differences between the HS-, GEL and TS-groups in hemodynamic parameters at Hb 3.0 g/dL.

Consistent with progressive hemodilution, DO_2_I further decreased in the remaining HS-, GEL- and TS-groups. In animals hemodiluted with hetastarch, O_2 _extraction was significantly increased as reflected by lower partial venous oxygen pressure (pvO_2_) (*P *< 0.05 vs. baseline and vs. GEL-group) and by a higher O_2 _extraction ratio (*P *< 0.05 vs. baseline and vs. GEL- and TS-groups).

#### Hemodilution to Hb 2.7 g/dL (that is, Hb_crit _of the GEL-group)

Hemodilution could be continued to Hb 2.7 g/dL in the GEL- and TS-groups. CI did not increase further, but was now maintained by an elevation of HR in both groups (*P *< 0.05 vs. baseline). The MAP and SVRI decreased slightly (non-significant vs. baseline). In both groups, the ITBVI was still higher than at baseline (*P *< 0.05). However, at Hb 2.7 g/dL, the ITBVI was higher and SVV was lower in the TS-group (*P *< 0.05 vs. GEL-group). In the GEL-group, the O_2 _extraction ratio (O_2_-ER) and EVLWI were elevated when compared with baseline (*P *< 0.05, see Figure 3). No further differences between the TS- and GEL-groups were observed in hemodynamic or O_2_-derived parameters at Hb 2.7 g/dL.

#### Hemodilution to Hb 2.1 g/dL (Hb_crit _of the TS-group)

Hemodilution could be performed most extensively with tetrastarch. When animals of the TS-group met their individual Hbcrit at 2.1 g/dL, the following changes were significantly different to baseline: HR, CI, SVI and EVLWI increased by 30%, 90%, 36% and 38%, while SVV, SVRI, DO_2_I, pvO_2 _and BE decreased by 52%, 64%, 40%, 15% and 72%, respectively (*P *< 0.05). Moreover, O_2_-ER increased by 38% and by 40% (see Table 3).

## Discussion

The main findings of the present study are that during acute normovolemic anemia, 1) the choice of the IV fluid has an impact on the maintenance of tissue oxygenation as reflected by variation in the extent of anemia tolerance; 2) anemia tolerance is higher when using colloids than when using crystalloids, and 3) among the colloids, tetrastarch (6% HES 130/0.4) provided higher anemia tolerance than did gelatin (3.5% urea-crosslinked polygeline) and hetastarch (6% HES 450/0.7). This was reflected by significantly lower values of Hb_crit _and a higher allowable volume of blood exchanged for tetrastarch.

Hb_crit _is the hemoglobin concentration associated with a critical limitation of O_2 _supply and hallmarks the ultimate tolerance limit of acute normovolemic anemia [10,11]. In our previous experimental studies, hemodilution to Hb_crit _was associated with 100% mortality, if no further treatment (such as elevation of fraction of inspired O_2_, transfusion of red blood cells, or infusion of artificial O_2 _carriers) was initiated after institution of critical normovolemic anemia [12-14]. Several authors found Hb_crit _at values between 1.6 and 3.0 g/dL [10,12,14-21]. In detail, Hb_crit _was reduced by 1) hypothermia (moderate reduction of body core temperature reduces total body O_2 _demand [15]); 2) hyperoxemia (bioavailability of physically dissolved O_2 _is excellent in profound anemia [12,16,17]); 3) infusion of norepinephrine (stabilization of coronary perfusion pressure during hemodilution [19]); 4) artificial O_2 _carriers (maintenance of arterial oxygen content despite reduced hematocrit [18,22]), and 5) con-tinuous neuromuscular blockade (lowering skeletal muscular O_2 _demand [23]).

While different infusion fluids were used for hemodilution in the aforementioned studies, their particular impact on the limit of anemia tolerance has not been fully elucidated. In a similar hemodilution study performed in anesthetized and splenectomised dogs, van der Linden and coworkers found no differences between pentastarch (6% HES 200/0.5) and a 3% gelatin preparation in relation to the value of Hb_crit _[20]. However, the typical increase of CI in compensation for dilutional anemia was absent in that study, which was explained by cardio-depressant effects of the anesthesia regimen employed [20,21].

In the present study, gelatin provided more extensive anemia tolerance than did hetastarch. While the occasional use of hetastarch is predominantly reported by US physicians [24], European physicians rather avoid this fluid due to its adverse effects on coagulation and renal function [25]. Nevertheless, a recent clinical study found a reduction of mortality in trauma victims resuscitated with hetastarch in addition to the advanced trauma life support (ATLS)-standard of care treatment (that is, crystalloids along with blood products) [26].

Hetastarch is a hyperoncotic infusion fluid with a high viscosity *in vitro *(see Table 1). However, the effects of these properties *in vivo *are still not fully understood. While a decrease of plasma viscosity entails the increase of venous return, thereby enabling the hemodynamic compensation of acute anemia [27], recent experimental data suggest that increased plasma viscosity prevents microvascular collapse (for example, after fluid resuscitation from hemorrhagic shock) [6]. Furthermore, the effect of hetastarch on plasma viscosity is limited by its oncotic properties, as the reabsorption of interstitial fluid involves the dilution of viscogenic materials thereby lowering plasma viscosity in the long term [28].

In the present study, animals hemodiluted with hetastarch featured a significantly higher O_2_-ER at Hb_crit _when compared with animals of the TS- or GEL-group, indicating that macro- and microhemodynamic compensation of anemia was completely exhausted. In other words, hetastarch failed to prevent microcirculatory collapse at an earlier stage of hemodilution than did tetrastarch or gelatin. Although plasma viscosity was not assessed in the present study, it may be assumed that a dilution-related fading of viscogenic potential might have contributed to this result.

Whether fluid resuscitation should be performed with crystalloids or colloids, has been a matter of controversy for decades, and the discussion is still open [29]. Although most crystalloid infusion fluids are plasma-isotonic, they cross the capillary membranes within 20 to 30 minutes of infusion, and most of the volume infused is shifted into the interstitium. To maintain normovolemia with crystalloids, it has been recom-mended to replace an acute blood loss in a ratio of at least 1:3 [30]. However, the exclusive use of crystalloids for volume replacement results in edema formation and may thereby compromise tissue oxygenation [31]. Actually, excessive tissue hydration due to pure crystalloid volume replacement in the perioperative phase was held responsible for many postoperative complications including increased incidence of anastomotic dehiscence in abdominal surgery, postoperative vomiting and orthostatic dysregulation [32,33]. Moreover, an experimental study in pigs subjected to colon anastomosis surgery found that microcirculatory blood flow and oxygen tension in perianastomotic colon tissue were increased in animals infused with tetrastarch when compared with crystalloid fluid management [34]. This finding was explained by homogenisation of mucosal microcirculatory blood flow after infusion of tetrastarch.

In the present study, with Ringer's solution, the exchange of blood was associated with increased pulmonary edema formation (elevated EVLWI) and an impairment of pulmonary gas exchange (decreased paO_2_). This phenomenon was not observed in animals hemodiluted with gelatin, hetastarch or tetrastarch. Although EVLWI merely reflects the degree of tissue hydration at the site of pulmonary circulation, it may be supposed that a relevant edema formation also occurred in peripheral O_2_-consuming tissues: while O_2 _extraction increased consistently in animals hemodiluted with any of these colloids, O_2 _extraction was not increased when animals of the RS-group met their individual Hb_crit_. As this finding may reflect a microcirculatory disorder related to excessive tissue hydration, we conclude that the comparatively early VO_2_-decrease in the RS-group might, in addition to the anemia-related restriction of the O_2 _transport capacity, also be attributable to an edema-related impairment of O_2 _uptake at the site of pulmonary and peripheral microcirculation.

A certain transcapillary filtration rate is also characteristic for gelatin preparations [35]. Their low molecular weight (30 to 40 kDa) entails a rapid passage into the interstitial space and a rapid clearance by glomerular filtration, finally reducing volume efficacy to 80% (that is, 20% extravasation rate). Consistently, EVLWI increased much later in the GEL-group than in the RS-group. Moreover, when hemodilution was continued below Hb 3.7 g/dL, O_2 _extraction could be augmented, reflecting that microcirculatory function might have been maintained more adequately than in the RS-group.

The strict maintenance of normovolemia is essential for adequate hemodynamic compensation of acute anemia, that is, for the increase in cardiac output. However, one weakness of the present protocol is the verification of normovolemia at time points characterized by extreme anemia. During extreme hemodilution, the kinetic of ICG elimination is significantly altered by dilution of albumin and increased cardiac output. Therefore, a measurement of circulating blood volume at this point would yield results not comparable with the baseline measurement [7], so normovolemia was deduced from clinically assessable parameters, for example, ITBVI and SVV.

While ITBVI represents LV preload, decreases in SVV reflect adequate volume responsiveness. After hemodilution with either fluid, ITBVI increased above the baseline level, indicating that volume replacement was adequate to achieve macro-hemodynamic compensation of acute anemia (increase of venous return to the heart, augmentation of LV preload). However, the increase of ITBVI was more pronounced in the TS- and HS-groups. In the course of hemodilution, SVV decreased in the TS-, GEL-, and HS-groups. In the RS-group, SVV remained unchanged and at Hb_crit_, it was significantly lower than in the HS-group. Both findings reflect that volume responsiveness was most strongly expressed in the starch groups. In the RS-group, however, volume responsiveness and volume efficacy were limited by partial extravasation despite infusion of 3 mL RS per mL blood withdrawn.

Although this procedure provided adequate LV preload for hemodynamic compensation of acute anemia, it may be argued that the infusion of higher volumes of RS (4 or 5 mL per mL blood withdrawn) might have elevated intravascular volume and might have improved volume responsiveness. On the other hand, extravasation and tissue hydration would have exacerbated and might have further impaired O_2 _uptake and tissue oxygenation.

Likewise, the volume effect of gelatin appeared to be limited by its short intravascular half-life and its extravasation tendency. No differences between the colloid groups were observed in LV preload or volume responsiveness until hemodilution to Hb 3.0 g/dL. However, after continuation of hemodilution to 2.7 g/dL, ITBVI tended to decrease and SVV to increase in the GEL-group, and both parameters differed significantly from the TS-group. Nevertheless, animals of the GEL-group still featured sufficient hemodynamic compensation of acute anemia (CI, SVI and ITBVI remained increased vs. baseline), which precludes that they were actually hypovolemic.

These findings indicate that 1) the infusion of 1.2 mL gelatin per mL blood withdrawn was adequate to maintain LV preload and volume responsiveness over a wide range of the hemodilution protocol; 2) in spite of this, the volume effect of gelatin could not be sustained until the end of the protocol, and 3) that extravasation and fading volume responsiveness might have compromised microvascular perfusion, which finally caused animals of the GEL-group to earlier achieve Hbcrit than the TS-group.

We chose Hb_crit _as the primary endpoint of the hemodilution protocol. In our previous studies, lactate concentrations began to increase 60 to 90 minutes after institution of Hb_crit _[12-14,19]. Of note, we employed the identical hemodilution protocol in the present study but animals were killed immediately after achievement of Hb_crit_, so that elevated lactate concentrations could not yet be expected.

In the present experimental model, the effects of the investigated infusion fluids became apparent when the O_2 _transport capacity was driven to its critical limit. Whether differences between the groups existed already at less severe degrees of anemia, is difficult to conclude.

However, a clinical investigation in healthy volunteers undergoing moderate hemodilution with different HES preparations (HES 130/0.4, HES 70/0.5 or HES 200/0.5) demonstrated that tetrastarch provided the most sustained increase of tissue O_2 _partial pressure (tpO_2_) [36].

In summary, our data suggest that the choice of the IV infusion fluid used for acellular volume replacement has an impact on the maintenance of O_2 _supply. Among the fluids investigated in the present study, tetrastarch (HES 130/0.4) provided the most extensive anemia tolerance when compared with gelatin, hetastarch or Ringer's solution. While the exact underlying mechanism remains to be elucidated, extravasation and formation of interstitial edema were associated with decreased anemia tolerance, indicating that the microcirculatory effects of the fluids investigated had a major impact on tissue oxygenation.

## Conclusions

During acellular treatment of an acute blood loss in lieu of allogeneic blood transfusions (for example, correction of hypovolemia, bridging an unexpected massive blood loss, or declining of transfusion for religious reasons), modern low-molecular tetrastarch preparations (such as HES 130/0.4) appear most suitable to maintain O_2 _supply over a wide range of levels of acute normovolemic anemia.

## Key messages

• The correction of hypovolemia with acellular fluids implies the dilution of the cell mass remaining in the vasculature (acute normovolemic anemia).

• Sustainment of normovolemia and nutritive microcirculatory blood flow has a major impact on the maintenance of tissue oxygenation and thereby on the tolerance of acute anemia.

• The fluids investigated in the present study (tetrastarch, gelatin, hetastarch and Ringer's Solution) differed in their volume effects and their extravasation rate and might thereby have influenced microcirculatory function.

• In profound anemia, modern low-molecular tetrastarch preparations were most advantageous to maintain normovolemia as well as tissue oxygenation.

## Abbreviations

ANOVA: analysis of variance; BE: base excess; BSA: body surface area; BVI: circulating blood volume (indexed to BSA); CaO_2_: arterial oxygen content; CI: cardiac index (cardiac output indexed to BSA); DO_2_: oxygen delivery; EVLWI: extravascular lung water (indexed to BSA); Hb_crit_: critical hemoglobin concentration; HES: hydroxyethyl starch; HR: heart rate; IV: intravenous; ICG: indocyaningreen; ITBVI: intrathoracic blood volume (indexed to BSA); MAP: mean arterial pressure; MPAP: mean pulmonary arterial pressure; O_2_-ER: oxygen extraction ratio; paO_2_: arterial oxygen partial pressure; PCWP: pulmonary capillary wedge pressure; pvO_2_: mixed-venous oxygen partial pressure; SVI: stroke volume (indexed to BSA); SVRI: systemic vascular resistance (indexed to BSA); SVV: stroke volume variation; VO_2_: total body oxygen consumption.

## Competing interests

The study was sponsored by a research grant from the Else Kröner-Fresenius-Foundation, Bad Homburg, Germany. The authors declare that they have no further competing interests.

## Authors' contributions

AP, SK, HK, OH and ML carried out the experiments. SK coordinated data acquisition during the experiments and built up the database. PS performed the statistical analysis. AP, BZ and OH conceived the study and participated in its design. BZ and OH helped to draft the manuscript. All authors read and approved the final manuscript.
